# 

**Published:** 1891-01

**Authors:** 


### LIST OF CONTRIBUTORS.

Billings, Frank S.
Breisacher, Leo.
Bryden, Williamson.
Clement, W. A.
Curtice, Cooper,
Dinwidde, R. R.
Edwards, Fred. H. P.
Fleming, George,
Francis, M.
Griffen, E. Gerald.
Harger, S. J, J.
Hellier, J. B.
[illegible]oskins, W. Horace.
[illegible]idekoper, R. S.
[illegible]ilson, J. C.
[illegible]ntington, George S.
[illegible]sme, A.
[illegible]ohnson, D. A.
King, B. F.
Law, James.
Lee, Daniel D.
Lyfford, C. C.
Magnin, Louis.
Marsh, J. Wallace.
Mayo, M. S.
McLaughlin, John A.
Meyers, J. C.
Michiner, J. C.
Miller, W. E. B.
Morris, Claude D.
Neher, Herbert.
O'Leary, J.
Paquin, Paul.
Pearson, Leonard.
Peters, Austin.
Ridge, W. H.
Rogers, Thomas, B.
Salmon, D. E.
Sheperd, E. H.
Stewart, S.
Stiles, C. W.
Sutton, J. Bland.
Vandeveer, George G.
Vulliamy, H.
Waugh, James A.
Weber. S. E.
Williams, W. L.
Winchester, J. F.

14/062

### INDEX VOLUME XII.

Abscess of Guttural Pouch.......... 620
A Dipping Vat for Sheep.......... 462
Age of the Horse, Ox, Dog and Other Domesticated Animals, 25, 78, 118, 173, 226, 282, 327, 377, 443, 623, 661
Age of the Sheep and Goat.......... 623
Animal Intelligence..........121, 388
Arsenic, Can A. Produce Paralysis of the Lower Jaw?.......... 30
Barren Mares.......... 587
Back Number Wanted.......... 92
Billings, The Untrustworthiness of the Reports of the Government. 415
Biology of the Cattle Tick.......... 313
Books and Pamphlets received......58, 155, 207, 264, 312, 361, 410, 474, 586
Bryden, Williamson, Shoulder Lump.......... 423
The Transatlantic Cattle Trade.......... 495
Camel, A Manual of the Disease of, etc.......... 407
Cannabis Indica in Colic.......... 222
Cattle Tick, Biology of.......... 313
Cattle Trade (Transatlantic), Regulation of.......... 495
Change Publication.......... 92
Clement, A. W., Pseudo Tuberculosis.......... 185
Diverticulum of the Oesophagus.......... 216
Nail Wound of the Foot.......... 277
Colic, Cannabis Indica in.......... 222
Comparative Anatomy of Domesticated Animals.......... 655
Committee Reports.......... 75
Comparative Pathology.......... 1
Curtice, Cooper, The Oxwarble of the Unitep States.......... 265
The Biology of the Cattle Tick.......... 313
Development of the Horseshoe, Historical.......... 363
Dicephalus-Bicollis Monstrosity.......... 336
Dinwidde, R. R., The Germ of Texas Fever.......... 115
Diverticulum of the Oesophagus.......... 216
El Dourine.......... 171
Emasculator.......... 77
Epidemic Influenza.......... 656
Extent of Reason in the Lower Animals.......... 137
Extra Uterine Gestation. A Criticism.......... 429
Edwards, H. P. Fred., Dicephalus-Bicollis Monstrosity.......... 336
Fistulous Opening from the Mouth.......... 606
Fleming, George, Does Meniere's Disease Occur in Horse?.......... 319
Francis, M., The Screw Worm.......... 16
German Review..........189, 243, 295, 346, 389, 464, 638

Germ of Texas Fever .......... .... ........... ............... .... 115
Griffin, Gerald E., Porrigo (Texas Mange) in Horse........................ 426
Gestation, Extra Uterine......... ....... ........................... 429
Gutteral Pouches, Abscess.......... ............................. ... 620
Hernia, Traumatic.......... .......... ........... ............. 326
Historical Development of the Horseshoe.... .......... ............ 363
Horseshoe, Historical Development of. ............................. 363
Hoskins, W. H., Uniform Veterinary Education.... ... ............ 209
Publication.................................... 528
Hyposulphite of Soda.................. .......... ............ 109, 348
Huidekoper, R. S., Age of the Horse, Ox, Dog and other Domesticated Animals.....25, 78, 118, 173, 226, 282, 327, 377, 443, 623, 661
Identification of Animals ........ .................... 610
Huilson, O. C., Thrombosis in Iliac Arteries.............................. 279
Hunt, V. G., Osteo Perosis..................................... 359
Identification of Animals.................... .................... 610
Iliac Arteries, Thrombosis in..................................... 279
Illinois State Veterinary Medical Association........................ 33
Indian Journal. ..................................... ................ 25
Influenza in Horses.............. ............................... 129
Inspection of Cattle and Sheep for Export.................... ........... 31
Intelligence, Animal.................................... ......... .... 121
Investigations of Animal Diseases............................... 415
Investigation of Swine Diseases.................................. 419
Iowa State Veterinary Medical Association......................... 33
Jasme, A., Osteo Porosis...............................307, 359, 404
King, B. F., Lymphatics........................................ 218
Koch's Lymph in Bovine Tuberculosis ................................ 181
Koch's Method with Tuberculosis................................. 58
Laryngeal Injections..................................... ..... 164
Leukaemia. ............. .................................. ....... 126
Locomotor Ataxia in the Horse................................... 333
Lymphatics............................................................. 218
Law, James, Koch's Method with Tuberculosis ........................ 51
Lee, D. D., A Peculiar Monstrosity................................. 8
Prevalence of Tuberculosis.............................. 274
Fistulous Opening from the Mouth....... ................ 606
Lyford, C. C. Barren Mares......................... .............. 587
Magnin, Louis, Locomotor Ataxia in the Horse...................... 333
Mallein, Recent Experiments of..................................... 411
Mange, Texas........................................................ 426
Marsh, Wallace, J., Hyposulphite of Soda, Traumatic Hernia..... 326, 348
Mayo, M. S., Tuberculosis in a Boar................................. 345
Measles in Horses ................................................ 609
Meniere's Disease, Occur in Horses?................................ 319
Meningitis........................................................ 167
Meyers, J. C. Tracheotomy and Laryngeal Injections in the Throat..... 164
Michener, J. C., Meningitis................................ 157, 344
Monstrosity Dicephalus Bicollis.................................... 336

Monstrosity, a Peculiar.................... 5
Morphine, How Much Can a Dog Stand?.................... 30
Morris, D. Claude, Tetanus.................... 439
Nail Wound of the Foot.................... 277
Necrology.................... 145

NECROLOGY.
Joseph Leidy.................... 308
Leon Lafosse.................... 310
Nostrand.................... 310

Neglected Opportunities. Agricultural Shows.................... 461
Neher, Herbert, Remedies, Cases.................... 325, 634
Oesophagus Diverticulum of.................... 216
Open Letter.................... 20
Oxwarble of the United States.................... 265
Paquin, Dr., Answer to Open Letter.................... 20
Paralysis produced by Arsenic.................... 30
Patents, Recent....................84, 141, 171, 232, 286, 337, 382, 631
Pearson, Leonard, Recent Experiment with Mallein.................... 411
Recent Foreign Investigation of Swine Diseases.................... 419
Peculiar Monstrosity.................... 8
Pennsylvania State Veterinary Medical Association....................125, 459
Peters, Austin, Intelligence and Education.................... 514
Pleuro-Pneumonia Contagiosa.................... 241
Pseudo Tuberculosis.................... 85
Purrigo (Texas Mange) in Horses.................... 426
Rachitis.................... 477
Reason Extent of, in Lower Animals.................... 137
Recent Experiments with Mallein. A Lymph made from Cultures of the Bacillus of Glanders.................... 411
Recent Foreign Investigations of Swine Diseases.................... 419
Recent Patents....................84, 141, 171, 232, 286, 337, 382, 631
Remedies.................... 325
Reviews.................... 187
Roaring (Hemiplegia Laryngis).................... 9
Ridge, W. H. H., El Dourine.................... 171
Rogers, Thomas B., Cases.................... 635
Salmon, D. E., Committee Report.................... 75
Screw Worm.................... 16
Sheperd, E. H., Hyposulphite of Soda.................... 109
Shoulder Lump.................... 423

SOCIETY PROCEEDINGS.
Alumni Association, American Veterinary College.................... 203
California State Veterinary Association.................... 147
Connecticut Veterinary Medical Association.................... 349
Illinois State Veterinary Medical Association....................33, 191
Indiana Association of Veterinary Graduates.................... 93
Kansas Veterinary Medical Association.................... 653
Keystone Veterinary Medical Association....................40, 102, 253, 305, 650

Long Island Veterinary Association........ ..................41, 102
Maryland State Veterinary Medical Society...................... 202
McGill University Association for the study of Comparative Psychology, in connection with the Faculty of Comparative Medicine and Veterinary Science for the years 1889-1890..... 34
Massachusetts Veterinary Association......44, 201, 248, 306, 350, 402
Nebraska Veterinary Medical Association..... ................. 201
New Jersey State Veterinary Society........................149, 475
New Jersey Veterinary Medical Association of................. 252
New York State Veterinary Society...................... . 151, 470
New York State Veterinary Medical Society..................... 345
Ohio State Veterinary Medical Association...................... 99
Ohio and Michigan State Veterinary, Joint Session ..... ....402, 468
Ontario Veterinary Medical Association....................105, 150
Pennsylvania State Veterinary Medical Association..........195, 646
Philadelphia Society of Veterinary Medicine .................. 654
South Dakota Veterinary Medical Association.................. 654
United States Veterinary Medical Association................150, 502
Western Iowa Veterinary Medical Association........... ...... 474
Western Pennsylvania Veterinary Medical Association........... 474
Wisconsin Society of Veterinary Graduates..................... 203

Sheep Dips................................................... 301
Spanish Review............................................... 247
Soda, Hyposulphite of........................................ 348
State of Wisconsin........................................... 146
Stewart, S., Cannabis Indica in Colic........................ 222
Sutton, J. Bland, Comparative Pathology...................... 1
Extra Uterine Gestation........................ ...... 429
Tetanus...................................................... 439
Texas Fever, Germ of......................................... 115
Thrombosis in Iliac Arteries....................... .......... 279
Tigress, Tuberculosis in..................................... 225
Tracheotomy and Laryngeal Injections in the Throat............. .. 164
Transatlantic Cattle Trade and its Regulations............... 495
Traumatic Hernia............................................. 326
Tuberculosis ................................................ 345
Tuberculosis in a Boar....................... ............ 345
Tuberculosis, Koch's Lymph in................................188, 298
Tuberculosis, Koch's Method with............................. 51
Tuberculosis, in Monkey................. ..................... 304
Tuberculosis, Prevalence of......... ........................ 27
Tuberculosis, Pseudo......................................... 185
Tuberculosis in Tigress ..................................... 225
Uniform Veterinary Education .................... ............ 209
Untrustworthiness of the Reports of the Government in relation to Investigations of Animal Diseases ............................. 415
Uterine Gestation ........................................... 429
Vandeveer, George, G., Leukaemia...... ............... ......... 126

VETERINARY COLLEGES.
Alumni Association of the American Veterinary College........ 358
American Veterinary College Commencement.................. 206
Chicago Veterinary College.............................. 357
Chicago Veterinary College Commencement.................. 206
Harvard University, Dept. of Veterinary Medicine.............. 357
Lahore Veterinary College.................................. 354
Montreal Veterinary College Commencement.................. 260
New York College of Veterinary Surgeons Commencement...... 258
Ontario Veterinary College..............................46, 358
Ontario Veterinary College Commencement.................. 261
United States Veterinary Medical Association........33, 344, 386, 459
University of Nebraska.................................. 240
University of Pa. Veterinary Department Commencement....... 367
Veterinary Post Mortem Examinations........................ 409
Veterinary Education, Uniform.............................. 209
Veterinary Legislation.................................... 290
Veterinary Surgery........................................ 311

Vulliamy, H., Animal Intelligence............................ 121
Weber, S. E., Historical Development of the Horseshoe............ 363
Williams, W. L., National & International Meat Inspection........ 529

### ILLUSTRATIONS, VOL. XII.

FIG. PAGE
1 An Ovarian Hydrocele (Human)............................ 3
2 Ovarian Hydrocele in a Mare............................ 4
3 Ovarian Pouch of a Mouse................................ 4
4 Ovarian Pouch of a Baboon................................ 5
5 Ovarian Pouch of a Porcupine............................ 6
6 Ovarian Hydrocele in a Guinea-Pig........................ 6
7 An Ovarian Hydrocele.................................... 7
8 A Monstrosity (Calf).................................... 8
9 Tampon Canula (Moeller)................................ 13
10 Larynx of a horse...................................... 14
11 Screw-worm Single egg, greatly enlarged.................. 18
12 " Bunch of eggs.................................. 18
13 " Larva.......................................... 18
14 " Pupa, or Chrysalis.............................. 18
15 " Pupa case, showing broken end where fly emerged... 18
16 " Side view of head and mouth parts................ 19
17 " Fly............................................ 19
18 Horse's Mouth, Fifteen Years.............................. 26
19 Horse's Mouth, Seventeen Years............................ 28
20 Haussman Emasculator.................................... 77

FIG. PAGE
21 Horse's Mouth, Nineteen Years ..... 68
22 Horse's Mouth, Twenty-one Years ..... 80
23 Horse's Mouth, Thirty Years ..... 82
24 Collar Pad ..... 84
25 Spreader for Gaiting Horses ..... 84
26 Toe Weight and Quarter-Boot Fastener ..... 85
27 Horse Training Harness ..... 85
28 Bridle ..... 85
29 Bridle Attachment ..... 86
30 Horseshoe ..... 86
32 Watering-Trough for Stock ..... 87
33 Device for Holding Horses ..... 87
34 Hoof-Pad ..... 87
35 Bit for Horses ..... 88
36 Cow-tail Holder ..... 88
37 Poultry-Crate ..... 88
38 Checkrein for Harness ..... 89
39 Automatic Stock-Watering Through ..... 89
40 Independent Check for Horses ..... 89
41 Halter ..... 90
42 Martingale Attachment for Harness ..... 90
43 Harness Saddle ..... 90
44 Harness ..... 91
45 Horse's Teeth ..... 119
46 Horse's Teeth ..... 120
47 Harness ..... 141
48 Curry Comb ..... 142
49 Crupper Fastening ..... 142
50 Tether ..... 142
51 Mane Holder for Horses ..... 143
52 Hoof Expander ..... 143
53 Milk Testing and Separating Machine ..... 143
54 Device for Watering Stock ..... 144
55 Controlling Gear for Draft-Horses ..... 144
56 Heel Expander for Horses ..... 145
57 Teeth ..... 173
58 Horse's Teeth ..... 174
59 Horse's Teeth ..... 175, 176, 177, 178, 179
60 Hames ..... 180
61 Horseshoe ..... 181
62 Tool for Dehorning Calves ..... 181
63 Horse Collar ..... 182
64 Hoof Expanded ..... 182
65 Hoof Expander and Frog Developer for Horses ..... 182
66 Horse Blanket ..... 183
67 Implement for Dehorning Cattle ..... 183
68 Cattle or Stock Car ..... 184
69 Ox-Yoke ..... 185
70 Horseshoeing-Rack ..... 185

FIG. PAGE
71 Horse's Teeth........................................228, 229, 231
72 Automatic Animal-Feeder........................................ 232
73 Apparatus for Watering Hogs........................................ 232
74 Hoof Spreading Horseshoe........................................ 232
75 Device for Controlling Horses........................................ 233
76 Device for Attaching Horseshoes........................................ 233
77 Horseshoe Gage........................................ 233
78 Animal Collar........................................ 234
79 Sheep Shears........................................ 234
80 Horse Training Blind........................................ 235
81 Horse Collar........................................ 235
82 Heel Expander for Horses........................................ 236
83 Watering Trough for Stock-Cars........................................ 236
84 Animal Chute........................................ 237
85 Horse Protector........................................ 237
86 Double Deck Stock-Car........................................ 238
87 Hoof-Pad........................................ 239
88 Hypoderma Lineata Villers........................................ 267
89 Hypoderma Bovis........................................ 268
90 Hypoderma Lineata Villers........................................ 268
91 Hypoderma Bonassi........................................ 269
92 Hypoderma Lineata........................................ 269
93 Hypoderma Bovis........................................ 269
94 Egg of Hypoderma Lineata........................................ 269
95 Larval Hypoderma Lineata in Egg shell........................................ 270
96 Hypoderma Lineata........................................ 270
97 Hypoderma Lineata........................................ 271
98 Hypoderma Lineata........................................ 272
99 Lower Molar Arch of a very Old Horse........................................ 282
100 Molar Arches (Right) of a very Old Horse........................................ 283
101 Irregular Wearing of Upper & Lower Molars........................................ 284
102 Hypertrophy of the Upper Fourth Molar........................................ 285
103 Watering Trough for Stock........................................ 286
104 Animal Poke........................................ 286
105 Foot Rasp for Horses........................................ 287
106 Horseshoe........................................ 287
107 Hopple........................................ 287
108 Cattle Dehorner........................................ 288
109 Toe Weight for Horses........................................ 288
110 Animal Yoke........................................ 288
111 Veterinary Inhaler........................................ 289
112 Attaching Toe-Weight for Horses........................................ 289
113 Horseshoe........................................ 289
114 Horse Tail Holder and Rein Guard........................................ 290
115 Syringe........................................ 326
116 Crib Marks by Pressure of the Teeth against foreign bodies........ 329
117 Crib Marks by seizing foreign bodies ; inside of the jaw........... 330
118 Crib Marks........................................ 331
119 Cribbing........................................ 332

FIG. PAGE
120 Nose Bag........ 337
121 Watering Device for Stock-Cars........ 338
122 Bridle-Bit........ 338
123 Dehorning Implement........ 338
124 Bridle........ 339
125 Rubber Pad for Interfering-Straps........ 339
126 Nose-Bag........ 339
127 Hitching Device for Animals........ 340
128 Animal-Poke........ 340
129 Veterinary Instrument........ 341
130 Nose Ring for Animals........ 341
131 Veterinary File........ 342
132 Anti-Cribbiting Device for Horses........ 342
133 Regulator for Feed Boxes........ 343
134 Nose-Band for Animals........ 343
135 Thin Iron Plates covering the whole hoof........ 365
136 Asiatic Cap-Iron-Sole........ 366
137 Roman Shoes........ 367
138 German, 15th Century Shoe........ 368
139 Samratsche Horse........ 369
140 Grecian and Montenegrin Horseshoe of the present........ 370
141 Old French Horseshoe........ 371
142 Plate-Like Horseshoe of Southern Character........ 375
143 Horse's Teeth........ 381
144 Horseshoe........ 382
145 Animal Stock........ 383
146 Leg Spreader for Horses........ 384
147 Veterinary Incisor Cutter........ 348
148 Bridle........ 385
149 Supplemental Horseshoe........ 385
150 Calf Weaner and Udder Protector........ 386
151 Incomplete Delivery in a Monkey........ 430
152 A Mumified Calf retained in the Uterus 16 months........ 431
153 Head and one of the Feet of a Lamb retained in the Uterus........ 432
154 Intra-Uterine Maceration of a retained Lamb........ 433
155 The Uterus of a Jackal........ 438
156 Left Pincher Teeth of the Ox........ 444
157 Incisors of Calf........ 448
158 Two Years Teeth of Ox........ 449
159 Two Years and Nine Months........ 450
160 Three Years Six Months Ox........ 451
161 Four Years, Teeth of Ox........ 452
162 Six, Eight, Ten, Eleven and Fifteen Years Ox........ 455
163 A Dipping Vat for Sheep,........ 463
164 Rachitis........ 491
166 Rachitis........ 493
167 Male Pelvis, Horses........ 589
168 Long. Sec. of the Free Extremity of the Horse's Penis, relaxed state 590
169 Long. Sec. of the Free Ext. of the Penis in an erect state........ 590

FIG, PAGE
170 Bladder and Interpelvic Portion of Urethra opened from below.... 591
171 Generative Organs of the Mare.................................. 591
172 Old Models, Pregnators.......................................... 604
173 New Models, Pregnators.......................................... 605
174 Fistulous opening from the Mouth................................ 607
175 Fistulous opening from the Mouth................................ 607
176 Fistulous opening from the Mouth................................ 608
177 Age of Sheep and Goat, External Face and Internal Face........ 627
178 Age of Sheep and Goat, External and Internal Face.............. 628
179 Horns, Merino Ram, Montenegrin Ram, Goat....................... 630
180 Removable Attachment for Horseshoes............................ 631
181 Dehorning Implement............................................. 631
182 Horseshoe....................................................... 631
183 Blanket Muzzle.................................................. 632
184 Veterinary Mouth-Opener......................................... 632
185 Interfering Boot for Horses..................................... 633
186 Anti-Interfering Device for Horses.............................. 633
187 Wearing-Pad for Horses.......................................... 633
188 Rectal Injection................................................ 635
189 Echinorhynchus Gigas, Female.................................... 657
190 Echinorhynchus Gigas, Male...................................... 658
191 Lower Jaw of Pig................................................ 662
192 Skull of Young Pig.............................................. 665
193 Skull of Young Pig.............................................. 668
194 Skull of Young Pig Diseased..................................... 668
195 Skull of Texas Boar............................................. 669
196 Skull of Yorkshire Sow.......................................... 669
197 Teeth of Dog.................................................... 672
198 Skull of Greyhound.............................................. 673
199 Skull of Pug.................................................... 673
200 Teeth of Young Dogs............................................. 675
201 Teeth of Dogs................................................... 676
202 Teeth of Dogs................................................... 677

### BOOKS AND PAMPHLETS RECEIVED.

*Antiguedades de Costa Rica por el Dr. H. Polakowsky, Anales del Museo Nacional.* San Jose, 1890.

Johns Hopkins University Circulars, December, 1890. Baltimore.

*The Canadian Record of Science.* Vol. iv, No. 4. Montreal.

*Le Naturaliste Canadien.* Cap Rouge.

